# *MALAT1*: A Long Non-Coding RNA with Multiple Functions and Its Role in Processes Associated with Fat Deposition

**DOI:** 10.3390/genes15040479

**Published:** 2024-04-10

**Authors:** Katarzyna Piórkowska, Karolina Zygmunt, Walter Hunter, Ksenia Wróblewska

**Affiliations:** 1 National Research Institute of Animal Production, Animal Molecular Biology, 31-047 Cracow, Poland; karolina.zygmunt@iz.edu.pl (K.Z.); ksenia.wroblewska@iz.edu.pl (K.W.); 2 Faculty of Biotechnology and Horticulture, University of Agriculture in Cracow, 31-120 Cracow, Poland; waltersamuelhunter@gmail.com

**Keywords:** lncRNA, *MALAT1*, cancer, cell cycle, fat deposition, adipogenesis

## Abstract

Metastasis-associated lung adenocarcinoma transcript 1 (*MALAT1*) belongs to the lncRNA molecules, which are involved in transcriptional and epigenetic regulation and the control of gene expression, including the mechanism of chromatin remodeling. *MALAT1* was first discovered during carcinogenesis in lung adenocarcinoma, hence its name. In humans, 66 of its isoforms have been identified, and in pigs, only 2 are predicted, for which information is available in Ensembl databases (Ensembl Release 111). *MALAT1* is expressed in numerous tissues, including adipose, adrenal gland, heart, kidney, liver, ovary, pancreas, sigmoid colon, small intestine, spleen, and testis. *MALAT1*, as an lncRNA, shows a wide range of functions. It is involved in the regulation of the cell cycle, where it has pro-proliferative effects and high cellular levels during the G1/S and mitotic (M) phases. Moreover, it is involved in invasion, metastasis, and angiogenesis, and it has a crucial function in alternative splicing during carcinogenesis. In addition, *MALAT1* plays a significant role in the processes of fat deposition and adipogenesis. The human adipose tissue stem cells, during differentiation into adipocytes, secrete *MALAT1* as one the most abundant lncRNAs in the exosomes. *MALAT1* expression in fat tissue is positively correlated with adipogenic *FABP4* and *LPL*. This lncRNA is involved in the regulation of PPARγ at the transcription stage, fatty acid metabolism, and insulin signaling. The wide range of *MALAT1* functions makes it an interesting target in studies searching for drugs to prevent obesity development in humans. In turn, in farm animals, it can be a source of selection markers to control the fat tissue content.

## 1. Long Non-Coding RNAs

Long non-coding RNAs are molecules longer than 200 nucleotides and are divided into intronic and intergenic ncRNAs, sense lncRNAs, anti-sense lncRNAs, enhancer-associated lncRNAs, and circular lncRNAs [1]. Previously, they were considered not to encode proteins, but it has recently been reported that most contain open reading frames and hat they are translated [2]. Early literature positions held that lncRNAs could be converted into small proteins or micro-peptides, but these peptides are often highly unstable structures and mostly lack biological functions [3]. However, recently, lncRNA-derived peptides have become hot topics owing to their functionality in carcinogenesis, cancer progression, and the immune response [4,5]. Meanwhile, regarding tissue specificity, numerous studies have suggested that lncRNAs are the most abundant in the testis [6] and neural tissue.

In 2017, the FANTOM5 project identified 28,000 lncRNAs when using different human sources [7]. Initial studies suggested that lncRNAs are highly conservative in their sequences in different species. They are poor in rare variants [8] and mutations of insertion/deletion types [9]. However, it was recently reported that lncRNAs are strongly conserved only at the genome stage and not at the transcript level, which means that they are not transcribed in the orthologous genomic region, which may be associated with rapid species-specific adaptive selection [10]. Studies of lncRNA function are still insufficient, although evidence shows that most lncRNAs in mammals are likely to be functional [6]. Nevertheless, their biological relevance has been presented for only a few species.

In humans, approximately 2600 lncRNAs have been annotated as functional, a number that is much lower in other vertebrate species [11]. In the literature, it is described that lncRNAs are involved in transcriptional and epigenetic regulation. Overall, they control gene expression and the cell cycle, including the mechanisms of chromatin remodeling [12] and miRNA sponging to relieve or inhibit the binding action of miRNA with target transcripts [13]. Finally, some lncRNAs expressed from enhancer or silencer regions can bind to target transcripts and enhance [14] or inhibit [15] their subsequent translation. Therefore, it can be said that these molecules are essential regulators of gene expression because they can change gene expression conditions at various molecular levels. Moreover, large-scale investigations considering numerous molecules provide evidence that lncRNAs are, in fact, peptide-coding [16]. Consequently, providing information about newly described lncRNAs and confirming their biological function using in vitro methods seem necessary, especially in animals, which are studied less frequently than humans.

## 2. Molecular Structure and Expression of *MALAT1*, a Highly Interesting lncRNA

*MALAT1* belongs to the lncRNA family and plays various roles in the regulation of gene expression. It was previously described in many species, including humans. It received the name metastasis-associated lung adenocarcinoma transcript 1 [17] because initially it was identified as a prognostic marker of poor outcomes in patients with early-stage non-small-cell lung cancer [18]. The *MALAT1* gene is also known as PRO1073, *NCRNA00047*, *HCN, NEAT2,* and *LINC00047* according to human gene nomenclature (https://www.genenames.org/data/gene-symbol-report/#!/hgnc_id/HGNC:29665, accessed on 15 November 2023). Moreover, in the literature, *MALAT1* is referred to as hepcarcin, nuclear enriched abundant transcript 2, nuclear paraspeckle assembly transcript 2 (non-protein coding), and long intergenic non-protein coding RNA 47 (HGNC:29665, NCBI Gene:378938). For humans, *MALAT1* is located on chromosome 11: at 65,497,640–65,508,073 on the forward strand (Ensembl). In the human *MALAT1* gene, 66 isoforms have been identified (GRCh38.p14, GCA_000001405.29, Ensembl Release 111, January 2024), and the longest isoform (*MALAT1*-201) contains 10,434 bp.

The genetic databases provide information about mouse *MALAT1* (https://www.informatics.jax.org/marker/, accessed on 15 November 2023, MGI:1919539) as well, according to GRCm39. In mice, the *MALAT1* gene is located on chromosome 19: at 5,845,717–5,852,706. To date, 21 isoforms of the *MALAT1* gene have been identified, the longest being Malat1-201 with 6988 bp. Studies using GMO mice identified five phenotypes associated with *MALAT1* lncRNAs based on a *MALAT1* KO study: abnormal nervous system physiology [19], brain inflammation [20], increased brain apoptosis [21], increased cerebral infarct size [19], and increased susceptibility to ischemic brain injury [22].

In a recent study, Piórkowska et al. [23] identified an lncRNA (ENSSSCG00000048856) within the subcutaneous fat transcriptome of pigs, which was differentially expressed between individuals with low and high subcutaneous fat deposition highly conserved with the human *MALAT1* gene. Porcine *MALAT1* was previously described by Yang et al. in 2017 [24], who found that the identified CUFF.253988.1 lncRNA shared homology with the human *MALAT1*. However, in Ensembl databases, this lncRNA still appears without names but as a novel gene. This gene in pigs is located on chromosome 2: at 6,751,519–6,757,180 encoded at the reverse strand (according to Sscrofa11.1, Ensembl Release 111, January 2024), and it is predicted that it has two isoforms, the longest being 3503 bp, but studies on *MALAT1* in pigs are few in number, so these observations need to be extended. The porcine genomic sequence of *MALAT1* reveals homology with the human *MALAT1* at 83% and the mouse *MALAT1* at 78% (Blast NCBI). Moreover, Piórkowska et al. [23], when testing the subcutaneous fat tissue transcriptome, concluded that porcine *MALAT1* has additional isoforms, which, for example, correspond to human *MALAT1-201*, the longest isoform. Therefore, the state of porcine *MALAT1* isoforms still seems to be unsolved.

Although *MALAT1* has a genomically encoded poly(A) tract, during post-transcriptional processing, the poly(A) tail is missing [25]. The authors described RNase P as cleaving the primary *MALAT1* transcript downstream of the genomically encoded polyA-rich tract to, in parallel, generate 3′ of mature *MALAT1* transcript and 5′ of small tRNA-like molecules. The resultant 3′ end of the nuclear *MALAT1* transcript post-processing is not polyadenylated, but it contains a genomically encoded poly(A)-rich stretch. The long *MALAT1* transcript is localized to nuclear speckles [26] and the small t-RNA-like *MALAT1* in the cytoplasm [25]. The small *MALAT1* molecule has a triple-helix structure [27], which is highly stable in cancer cells and less stable in other cell cultures [28]. This triple-helical structure confers stability and nuclear localization in the absence of a true polyA tail. Moreover, *MALAT1* is known to be misregulated in many human cancers [29].

*MALAT1* is expressed in numerous tissues according to the ENCODE project [30], with high abundance in the adipose tissue, adrenal gland, heart, kidney, liver, ovary, pancreas, sigmoid colon, small intestine, and spleen; less in the testis and lung; and low expression in the brain. The Roslin Institute investigated the transcriptome of male and female pigs and identified enriched *MALAT1* expression in numerous tissues (https://www.ensembl.org/Sus_scrofa/Gene/ExpressionAtlas?db=core, accessed on 15 November 2023; g = ENSSSCG00000048856; r = 2:6751519-6757180). Moreover, Piórkowska et al. [23] showed that porcine *MALAT1* expression in subcutaneous fat tissue was positively correlated with the thickness of backfat, making *MALAT1* highly interesting in the context of adipogenesis and fat-deposition-related processes.

## 3. Upstream Regulation of *MALAT1* Gene Expression

*MALAT1* lncRNA was thoroughly investigated in the context of cancer prognosis/entities and metastasis, so examples of the upstream regulation of *MALAT1* transcription are tightly associated with cancer-related processes. In hepatocellular carcinoma (HCC), Huang et al. [31] observed that the expression of transcription factors Sp1 and Sp3 correlated with *MALAT1* expression, and the co-silencing of both TFs repressed transcription of this lncRNA, which highlights the positive regulation of *MALAT1* expression by these transcription factors. In a study analyzing *PCDH10* function in the context of tumor suppression, Zhao et al. [32] observed that overexpression of *PCDH10* in *AN3CA* and *HEC-1-B* cell lines significantly downregulated *MALAT1* expression, which was correlated with cell proliferation. The authors proved that this suppression is mediated by the canonical Wnt/β-catenin signaling pathway. Moreover, a study of mice reported that *MALAT1* expression was induced by hypoxia [33], and further analysis identified that this condition and its regulation are involved in the CaMKK/AMPK/HIF-1α axis [34], which is strongly associated with Ca^2+^ inputs for the augmentation of the *MALAT1* promoter during hypoxia. Moreover, it was observed that during the malignant transformation of human hepatic epithelial cells induced by arsenite, *MALAT1* and hypoxia-inducible factor (HIF)-2α created a feedback loop, because *MALAT1* causes dissociation of von Hippel-Lindau (VHL) protein from HIF-2α, which leads to the accumulation of this protein, and then HIF-2α regulates the transcription of *MALAT1* [35]. In turn, during oxidative stress conditions in endothelial cells under H2O2 exposure, it was found that *MALAT1* transcription could be induced by the p53 protein [36], the regulation of which in mice was previously suggested [37].

In other cancer research aiming to develop a therapeutic target for the treatment of Ewing sarcoma (EWS), it was found that *MALAT1* transcription was dependent on spleen tyrosine kinase (SYK)-mediated signaling, and c-MYC TF promoting SYK’s binding to the *MALAT1* promoter, which enhanced the proliferation of EWS [38]. In addition, in colorectal and gastric (GC) cancer cultures, it was observed that silencing of Yes-associated protein 1 (YAP1), which plays a significant role in the development of numerous carcinomas, led to the downregulation of *MALAT1* expression [39]. The role of *MALAT1* in therapy for multiple myeloma (MM) was investigated by Amodio et al. [40], who determined that *MALAT1* is entangled in a positive feedback loop with *NRF1* and *NRF2* TFs modulated by *KEAP1*, which suggests that targeting *MALAT1* will offer a novel powerful option for the treatment of MM. NRF1 is a key regulator of the proteasome bounce-back response, and its inhibition sensitizes cancer cells to proteasome inhibitors [41]. In turn, proteasome inhibitors are believed to be promising drugs for the treatment of proteasome-activated cancers such as MM [42]. Meanwhile, NRF2 has been shown to be associated with the malignant phenotype among all myeloma cells [43]. In a study investigating the influence of the SOX17 protein in esophageal squamous cell carcinoma (ESCC), it was observed that human *MALAT1* contains an SRY element in its promoter, which is associated with SOX17 via TF binding [44], and then the authors suggested that SOX17 significantly limits *MALAT1* expression. Moreover, in the same cancer entity, it was observed that, post-transcriptionally, *MALAT1* molecules can be regulated by miR-101 and miR-217 [45], leading to *MALAT1* silencing and suppressing the proliferation of ESCC cells by arresting the G2/M cell cycle. In turn, Koshimizu et al. [46] reported that during neuroblastoma development, *MALAT1* expression is sensitive to the activation of oxytocin cell surface receptors, and this induction of gene expression probably occurs through the cyclic AMP-responsive element binding (*CREB*) transcription factor, the binding site for which was identified in the *MALAT1* promoter. Furthermore, during bladder cancer, *MALAT1* was upregulated by TGF-β, which promotes tumor invasion and metastasis [47], and targeted inhibition of *MALAT1* suppressed the migration and invasion properties of TGF-β.

*MALAT1* expression and upregulation during liver regeneration were also investigated [48], and it was concluded that *MALAT1* plays a significant role in accelerating cell cycle progression in hepatocytes and promoting proliferation in vitro. It was observed that the hepatocyte growth factor increased *MALAT1* expression and that the p53 TF was involved in the negative regulation of *MALAT1* during liver regeneration. In other studies, *MALAT1* expression was stimulated in the kidneys of diabetic mice by a high increase in glucose, which is positively related to serum creatinine and urinary albumin levels [49,50]. Returning to post-transcriptional regulation, Leucci et al. [51] used the L428 and U87MG cell lines to prove that miR-9 regulates *MALAT1* expression mediated by AGO2.

The downstream regulations of *MALAT1* are described further in this paper, considering its significant role in numerous crucial processes.

## 4. *MALAT1* in Cell Cycle Regulation

The correct course of the cell cycle leading to the duplication of genetic information is the basis for maintaining cellular homeostasis, and its regulation, mainly through checkpoint pathways, includes cell quiescence, proliferation, and apoptosis [52]. Many studies have focused on examining the impacts of the expression levels of specific genes, including *MALAT1*, on the regulation of the cell cycle, mainly in the process of carcinogenesis, which involves uncontrolled proliferation and inhibition of the apoptosis of cancer cells. Numerous studies have shown that *MALAT1* has pro-proliferative effects, and high cellular levels of *MALAT1* have been observed during the G1/S and mitotic (M) phases [53,54]. Silencing *MALAT1* activity via two microRNAs, miR-101 and miR-217, as previously mentioned, leads to cell cycle arrest in the G2/M phase, probably through changes in the expression of p21, p27, and B-MYB [45]. Previously, BrdU-PI flow cytometry analysis showed that *MALAT1* depletion resulted in reduced replication and increased expression of the p53, p21, p27, and cyclin-2 genes, which are key to cell cycle inhibition. Subsequently, gene expression analysis of human diploid fibroblasts (HDFs) with *MALAT1* knockout showed reduced expression of genes involved in the transition from the G1 to the S phase (*CCNA2*, *CDC25C*, *Cdk1*, *E2F2*, and *MCM6*), replicative progression (*Cdc45*, *Cdt1*, *GINS2*, *GMNN*, *MCM3*, and *MCM10*), and mitotic progression (such as *AURKA*, *AURKB*, *BIRC5*, and *BUB1*) [53]. Similarly, knockout of the *MALAT1* gene resulted in a delay in the transition from the G1 to the S phase in LNCaP cells and reduced expression of the cyclin D1 (CCDN1) and CDK6 proteins, which are important at the G1/S restriction point [55]. Furthermore, the depletion of *MALAT1* resulted in decreased levels of B-MYB, which localizes to the promoters of genes that are expressed during the M phase, resulting in the aberrant expression of these genes [56]. Moreover, the interaction of *MALAT1* with the nuclear protein hnRNP C has been shown to support the translocation of *MALAT1* from the nucleus to the cytoplasm, promoting the transition from the G2 to the M phase [57]. On the other hand, many studies have indicated the role of *MALAT1* in the resistance of cancer cells to chemotherapy in chronic myeloid leukemia, head and neck squamous cell carcinoma, and hepatocellular carcinoma [58], increasing the ability to repair DNA, evade cell cycle checkpoints, and regulate apoptosis, autophagy, and stemness of cancer cells. Moreover, *MALAT1* stimulates the cell cycle of liver cells. In this tissue, knockout of *MALAT1* causes prolongation of the G0/G1 phase, and its overexpression resulted in an increased number of cells in the replication phase and a decreased number in the G0/G1 phase [48].

**Figure 1:**
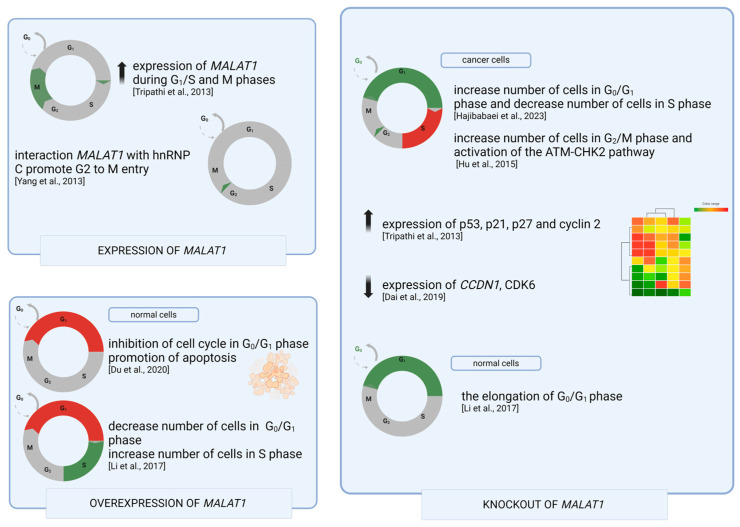
Role of *MALAT1* in the cell cycle of normal and cancer cells in cases of normal expression, overexpression, and knockout of *MALAT1*. Illustration was prepared in Biorender. [48,53,55,57,59,60,61].

However, Du et al. [59] reported that *MALAT1* overexpression inhibits the cell cycle in the G0/G1 phase and promotes apoptosis in endothelial cells. Moreover, flow cytometry of breast cancer (BC) cells with *MALAT1* knockout showed an increased number of cells in the G0/G1 phase, with a simultaneous decrease in the number of cells in the S phase [60]. The knockout of the *MALAT1* gene in esophageal cancer cells (ESCCs) resulted in an increased number of cells in the G2/M phase and activation of the ATM-CHK2 pathway, the role of which is to prevent too-rapid tumor growth by inhibiting the G2/M phase [61] (Figure 1).

Changes in the course of the cell cycle due to different expression levels of *MALAT1* depend on the type of cell. Cancer cells are characterized by an uncontrolled cell cycle as a result of mutations in tumor suppressor genes’ or oncogenes’ activity, as opposed to normal cells. Moreover, the type of cancer or the unique combination of genetic changes may provide different explanations of the impact of *MALAT1* expression on cell cycle changes.

## 5. Diseases Related to the *MALAT1* Gene

Though most of the disease entities related to *MALAT1* are associated with different kinds of cancers, it is also discussed in other disorders, such as liver fibrosis or diabetes.

Many studies have focused on cancers that occur in women, such as vulvar squamous cell carcinoma (VSCC), endometrioid endometrial carcinoma (EEC), epithelial ovarian cancer (EOC), and breast cancer. Increased expression of *MALAT1* has been confirmed in lobular breast cancer [62]. In EOC, increased *MALAT1* expression levels have been associated with distant metastases, and many studies indicate that *MALAT1* is involved in the process of metastasis [63]. It was shown that HPV-positive cells expressed *MALAT1*, but tissues affected by VSCC, a disease correlated with HPV, showed reduced levels of *MALAT1* expression [62,63]. In the course of EEC, the *PCDH10*–Wnt/β-catenin–*MALAT1* regulatory axis is a crucial component, and protocadherin 10 (PCDH10), a tumor suppressor gene, decreases the level of *MALAT1*, which is correlated with the inhibition of tumorigenesis [32]. *MALAT1* interacting with miR-218 was found to promote the proliferation of human choriocarcinoma cells by regulating the function of oncogenic F-box/WD repeat-containing protein 8 (Fbxw8) in phosphorylation-dependent ubiquitination [64].

Furthermore, numerous studies have focused on cancers of the digestive system, such as gastric cancer (GC) and colorectal cancer (CRC). The stimulatory effect of *MALAT1* on cancer cell proliferation in GC has been demonstrated [65]; moreover, Qi et al. [66] reported that *MALAT1* inhibits the anti-oncogene PCDH10, stimulating the proliferation of stomach cancer cells and metastasis. In the course of CRC, *MALAT1* stimulates the proliferation, invasion, and migration of cancer cells through PRKA kinase anchor protein 9 (*AKAP-9*), which is associated with cancer progression and metastasis [67]. The knockout of *MALAT1* was found to inhibit the proliferation of human tongue cancer cells and the metastasis process and increase the level of miR-124, which regulates the expression of jagged1 (*JAG1*), which, in turn, stimulates the proliferation of cancer cells [68].

Increased expression of *MALAT1* in the course of hilar cholangiocarcinoma (HCCA) was correlated with the pathological T stage of the tumor (according to the TNM classification), nerve invasion, and increased tumor area. Moreover, it was established that *MALAT1* is involved in oncogenesis by regulating CXCR4, which depends on mir-204 [69]. Upregulation of *MALAT1* has also been observed in other types of cancer, such as bladder epithelial cancer, with high malignant potential [70]. Increased *MALAT1* expression was demonstrated in infantile hemangioma (IH). Moreover, knockdown of *MALAT1* induced the process of apoptosis and cell cycle arrest in the replication phase in human umbilical vein endothelial cells (HUVECs) [71]. Increased *MALAT1* expression was demonstrated in acute myeloid leukemia (AML) and chronic myeloid leukemia (CML), and knockout of *MALAT1* in AML was found to inhibit proliferation by arresting the cell cycle in the G0/G1 phase and promote apoptosis of cancer cells by increasing the expression of caspase-3, -8, and -9 proteins [58,72].

However, changes in *MALAT1* expression have also been observed in other types of diseases, such as hyperglycemia, diabetic retinopathy, proliferative vitreoretinopathy, liver fibrosis, and hypertension. *MALAT1* regulates the expression of the cytokines interleukin 6 (IL-6) and tumor necrosis factor-alfa (TNF-α) by activating serum amplitude antigen 3 (*SAA3*) in arterial hyperglycemia [73]. Furthermore, increased expression of *MALAT1* has been demonstrated in RF/6A hyperglycemic model cells and the aqueous humor and fibrovascular membranes of patients suffering from diabetic retinopathy, a common complication of diabetes [74]. Increased *MALAT1* expression has also been observed in blood and fibrovascular membranes in another eye disease, proliferative vitreoretinopathy [75]. *MALAT1* overexpression promotes lipid accumulation, hepatic steatosis, and insulin resistance by increasing the expression of SREBP-1c, a sterol regulatory binding protein that is activated mainly by insulin, and the target genes *ACC1*, *ACLY*, *SCD1*, and *FAS* [76]. Increased *MALAT1* expression in liver fibrosis and regulation of botulinum C3 substrate 1 (Rac1) expression by miR-101b have been demonstrated to affect the cell cycle and activation of primary hepatic stellate cells.

On the other hand, it was found that the single nucleotide polymorphism rs619586A>G in *MALAT1* is associated with the risk of pulmonary arterial hypertension, increasing the expression of X-box binding protein 1 (*XBP1*), which inhibits the proliferation of vascular endothelial cells [77].

## 6. *MALAT1* Is Involved in Molecular Process Related to Carcinogenesis and Cancer Progression

*MALAT1*’s role in cancer biology is performed via the regulation of important signaling pathways that are involved in rudimentary processes like cell division or maintaining cell identity. As the full name of *MALAT1* suggests, this lncRNA influences cancer metastasis. One of the related pathway modes of action of *MALAT1* in metastasis is the Wnt/β-catenin pathway, which is activated by *MALAT1* overexpression. In detail, the 3′ end of *MALAT1* directly interacts with the N-terminal of EZH2 [78], thereby increasing its expression [78,79]. The upregulated EZH2 promotes GSK-3β downregulation by lysine 27 tri-methylation of histone 3. Through GSK-3β downregulation, β-catenin ubiquitination is inhibited, and the Wnt/β-catenin pathway is thereby activated [79]. However, in bladder cancer, researchers indicate that *MALAT1* interacts with suz12, another PRC2 component, instead of EZH2 [47]. In NK and T cell lymphomas, in turn, *MALAT1* was shown to interact with both PRC2 components [80]. Bioinformatic analysis of publicly available breast cancer datasets of protein–DNA and ncRNA–DNA binding interactions has identified 1293 genomics regions shared by PRC2 and *MALAT1*. The importance of the interaction was highlighted by GO enrichment analysis results, as the identified regions proved to be related to cancer malignancy [81]. Liang et al. [82] have shown that blocking Wnt/β-catenin, while simultaneously overexpressing *MALAT1,* abolishes the effects seen in a transwell migration assay and changes the expression profile of EMT markers typical for the mesenchymal phenotype. Apart from EMT markers’ regulation, the activation of the Wnt/β-catenin path by *MALAT1* leads to the upregulation of genes like c-Myc, cyclinD1, MMP-7, and CD44 [83], which are also linked to metastasis [83,84,85,86]. Another pathway related to *MALAT1* in the metastasis context is PI3K-AKT. Jin et al. [87] have found that by activating the PI3K-AKT pathway, *MALAT1* regulates EMT markers’ expression, namely E-cadherin, N-cadherin, vimentin, snail42, MMP2, and MMP9. The mechanism by which *MALAT1* influences the PI3K-AKT pathway has been described by Peng et al. [88], who demonstrated that *MALAT1* lowers the expression of miR-146a by sponging, which, in turn, downregulates the pathway by targeting the 3′UTR of *PI3K* mRNA.

Beyond metastasis, *MALAT1* has been implicated in apoptosis inhibition as a regulator of apoptotic-related proteins, both pro-apoptotic as well as anti-apoptotic, namely caspase-3, -8, and -9, Bax, Bcl-2, Mcl-1, and Bcl-xL [89,90,91]. The described mode of action again includes PI3K-AKT pathway activation [88], as well as interactions with microRNAs, like miR-101 and miR-125b [92,93]. For example, *MALAT1* binds to miR-140-5p, leading to the upregulation of *HDAC4*, an epigenetic regulator that was shown to inhibit transcription of the pro-apoptotic genes through chromatin modifications affecting corresponding promoters [94].

Wang et al. [95] imply that the cisplatin chemoresistance of oral squamous cell carcinoma cells is promoted by *MALAT1*-induced activation of PI3K-AKT-mTOR, which leads to growth factors’ upregulation, like VEGF, c-myc, and survivin [21]. In agreement with the above, the levels of VEGF, as well as another pro-angiogenic factor, FGF2, were found to be increased by the MALAT-1/mTOR/HIF-1α pathway, thereby promoting angiogenesis and ipso facto tumor growth [96].

Alternative splicing events that take place in cancer cells differ from those in healthy cells. Apart from splicing factors (SFs) like, for example, the serine-/arginine-rich family of nuclear phosphoproteins (SRs) or heterogeneous nuclear ribonucleoparticle proteins (HnRNPs), alternative splicing (AS) is also influenced by lncRNAs, for instance, *DGCR5* or *LINC01232*. An lncRNA can interact with an SF to protect them from degradation, direct their transport, or maintain interactions between different types of SFs. The *MALAT1* abundance in nuclear speckles, that is, the locations within the nucleus where splicing factors are assembled, suggests that this lncRNA is somehow significant in splicing events [97]. Indeed, *MALAT1,* like other lncRNAs, has been shown to be involved in AS by interacting with SFs to favor splicing variants that promote cancer development due to anti-apoptotic (BIM, BIN1) and pro-proliferative properties (TEAD1) [98]. The role of *MALAT1* was shown to be necessary for two splicing factors to bind—PTBP1 with PSF. The interaction between the three above-mentioned elements was found to be interdependent for the generation of a malignant phenotype (cell growth, invasion, migration) in hepatocellular carcinoma cells [99].

*MALAT1* overexpression is linked with a poor cancer prognosis, including in breast cancer [100], non-small-cell lung cancer [101], and glioma [102]. Accordingly, the *MALAT1* polymorphisms have been examined in regard to cancer risk contribution. Among the SNPs, there are cancer-risk-susceptibility as well as protective variants, as was demonstrated, for instance, in gastric cancer [103], papillary thyroid cancer [104], and hepatocellular carcinoma [105], as well as for cancer generally [106]. The correlation between specific polymorphisms and cancer risk might be explained by the alternations in *MALAT1* expression. In the rs664589 variant, the G allele impacts miR-194-5p binding and, in the aftermath, *MALAT1* degradation by Ago2 is inhibited, leading to its upregulation [107].

Overexpression of *MALAT1* often contributes to a wide array of changes within the cell. The mode of *MALAT1* action in cancer is multifold since it interacts with an array of microRNAs, as well as with mRNA and proteins, leading to significant changes within signaling pathways that, in turn, more or less contribute to the cancer phenotype. That being so, *MALAT1* should be highlighted in further research, as it emerges as a promising target for cancer therapies, including gene therapies.

## 7. Role of *MALAT1* in Processes Associated with Fat Deposition and Adipogenesis

Adipose tissue is a special type of connective tissue that is dominated by fat cells (adipocytes). Adipocytes can occur either singly or in groups in the connective tissue, but most often the cells come together in large clusters to form adipose tissue distributed throughout the body. An excessive accumulation of adipose tissue can lead to various metabolic diseases [108].

Obesity is a very strong factor that can predispose an individual to cardiovascular disease [109], diabetes [110], hypertension [111], and cancer [112]. The obesity epidemic is occurring not only in developed countries but also in developing countries. Obesity results from an imbalance between energy intake and consumption. Recently, research and scientific interest has been increasingly focused on the possible role of lncRNAs in obesity [113].

These studies attempt to explain the role of lncRNAs in obesity-related and fat deposition (FD) processes in humans and animals. Sun et al. [114] identified 1932 lncRNAs in adipose tissue (AT) and suggested that lnc_000414 is related to fat synthesis by inhibiting the proliferation of intracellular adipocytes. Another research team compared the backfat of Duroc and Chinese Luchuan pigs and found lncRNAs associated with 13 AT-related quantitative trait loci [115]. A recent study indicated that *LncIMF4* controls adipogenesis in intramuscular preadipocytes by relieving autophagy to inhibit lipolysis [116]. In turn, our previous study showed that one lncRNA, *MALAT1*, selected based on differentially expressed gene analysis, may be a potential regulator of processes related to fat deposition, which we surmised because it showed an increased expression in porcine AT, which was dependent on backfat accumulation [23]. In this study, we identified eight DE lncRNAs. However, orthologs for only two (*MALAT1* and *GLIS1*) were found in other species (BLAST analysis), while the rest were pig-specific.

**Figure 2:**
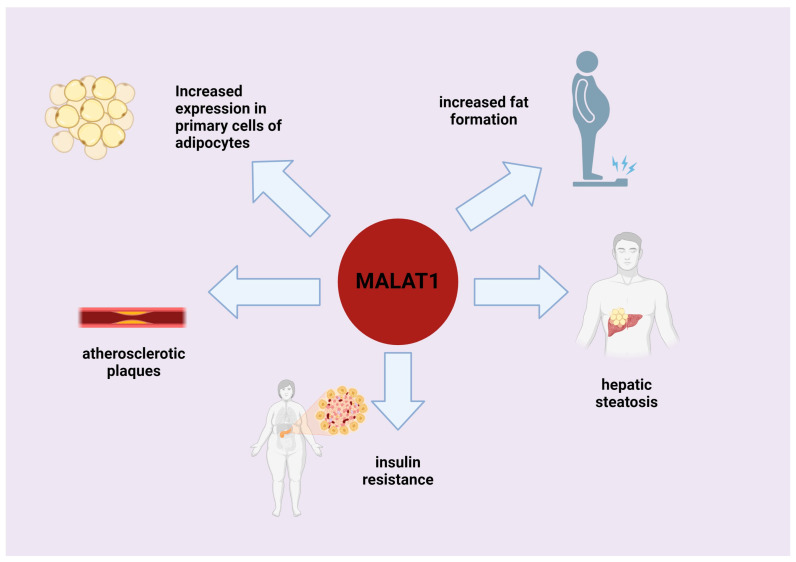
Role of *MALAT1* lncRNAs in fat tissue generation and deposition. Illustration was prepared in Biorender.

The data in the literature are diverse and controversial regarding the role of *MALAT1* in obesity and related disorders [117] (Figure 2). *MALAT1* is one of the most abundant lncRNAs identified in the exosomes of human adipose-tissue-derived stem cells (hADSCs). When hADSCs begin to differentiate into adipocytes, most *MALAT1* lncRNA is retained by preadipocytes and adipocytes [118]. Researchers have observed a significant reduction in *MALAT1* in visceral white adipose tissue (vWAT) in aged mice [117]. However, the significance of this lncRNA in adipose tissue remains uncertain.

*MALAT1* is a highly expressed lncRNA that affects the regulation of various physiological and pathological processes in many tissues. Various studies, and especially [119], have found that *MALAT1* is mainly localized in the nucleus, and in patients with cancer-related cachexia, *MALAT1* is downregulated in white adipose tissue (WAT) nuclei, which is associated with a low fat mass and a poor prognosis for cancer [119]. Other experiments have shown that *MALAT1* expression in fat tissue is positively correlated with the expression of the *FABP4* and *LPL* regulatory genes. These data indicate that *MALAT1* causes and enhances fat tissue formation. In addition, *MALAT1* regulates *PPARγ* gene expression and participates in adipogenesis at the transcriptional level through the PPAR signaling pathway, and it participates in fatty acid metabolism and insulin signaling [119].

Previous studies showed that *MALAT1* expression was lower in subcutaneous adipose tissue in obese mice [117], while higher levels of *MALAT1* were observed in adipose-tissue-derived stem cells from obese animals [120]. In contrast, more recent studies indicate that the level of *MALAT1* is reduced in the white adipose tissue of obese mice, while its deletion has neither a stimulatory nor inhibitory effect on diet-induced fat gain and lipid homeostasis in obese mice [121]. A positive correlation between *MALAT1* gene expression and insulin resistance was observed in the subcutaneous adipose tissue (SAT) of patients, suggesting the involvement of lncRNAs in the pathogenesis of obesity [121]. However, the transcription levels of *MALAT1* and *TUG1* showed a positive correlation with major lipogenic and adipogenic genes [121], and thus the authors suggested possible roles of *MALAT1* and *TUG1* in obesity. Moreover, Rasaei et al. [122] suggested that there may be a positive interaction effect between *MALAT1* transcription levels and the cholesterol/saturated fat index, which impacts the visceral adiposity index and body adiposity index among overweight and obese women. However, it was found that *MALAT1* expression was significantly reduced in atherosclerotic plaques. Furthermore, *MALAT1* inhibition significantly reduced the mRNA of *APOE* and *ABCA1* and increased ox-LDL uptake, lipid accumulation, and total cholesterol in macrophages. The authors also suggested that *MALAT1* may promote cholesterol accumulation by regulating the miR-17-5p/ABCA1 axis in ox-LDL-induced THP-1 macrophages [123].

Aging leads to dysregulation and partitioning of fat stores as well as insulin resistance, but the exact mechanisms involved in these phenomena remain unknown. *MALAT1* has been shown to be a gene that is strongly downregulated with aging, which may be due to a lower transcription rate and/or increased RNA instability during aging. Carter et al. [117] studied *MALAT1* RNA as a potent gene that is downregulated in vWAT during normal aging in male mice. In female mice, reduced levels of *MALAT1* in subcutaneous WAT (scWAT) were attributed to aging. In contrast, in males, a significant reduction in *MALAT1* expression levels in vWAT, but not scWAT, was observed with age. The effects of *MALAT1* on the size and number of adipocytes in WAT depots were investigated because *MALAT1* has been linked to the transition of the proliferation/differentiation state. The researchers detected that in male mice, similar levels of *MALAT1* were expressed in adipocytes and the stromal vascular fraction (SVF) in vWAT and scWAT. In vWAT of male mice, an age-related decrease in *MALAT1* levels was observed in adipocytes, but not in SVF. Moreover, reduced *MALAT1* expression in scWAT was also observed in genes (ob and db), as well as diet-induced obesity models. Based on these findings regarding *MALAT1+/+* and *MALAT1−/−*, Carter et al. [117] studied mice from a single litter to determine whether loss of *MALAT1* would affect age- or diet-induced fat mass gain and the development of glucose intolerance. The study found that Malat1-deficient males and females gained the same amount of weight and developed insulin resistance to a similar degree as *MALAT1+/+* mice. Mice from one litter were divided into two groups: one that received regular food and one that had a high-fat, sucrose-rich diet. The researchers observed no clear differences in oxygen consumption, food intake, or lipid profiles between *MALAT+/+* and *MALAT1−/−* mice, which indicates that the lack of *MALAT1* does not impair or accelerate age-induced fat gain and insulin resistance.

Moreover, *MALAT1* is responsible for fat accumulation in various organs. Its transcript has been shown to regulate lipid accumulation in the liver by increasing the stability of the sterol regulatory element-binding protein (SREBP)-1c [124]; this protein preferentially enhances the transcription of genes necessary for fatty acid synthesis. This thesis was supported by a study reported by Yan et al. [7], in which *MALAT1* levels were found to be increased in hepatic HepG2 cells and primary mouse hepatocytes treated with palmitate. Under palmitate treatment, the increase in *MALAT1* expression coincided with an increase in SREBP-1c in liver cells. The study showed that *MALAT1* knockout in mice significantly reduced liver lipid levels in vivo, while *MALAT1* overexpression in palmitate-treated HepG2 cells increased lipid accumulation. The study showed that excess palmitate increased *MALAT1* lncRNA expression, activated SREBP-1c, and induced intracellular lipid accumulation in hepatocytes. *MALAT1* expression was increased in hepatocytes exposed to palmitate and livers of ob/ob mice. The increased expression of *SREBP-1c* effectively abolishes the increase in intracellular triglyceride and cholesterol levels induced by *MALAT1*. This finding indicates that the effect of *MALAT1* on intracellular lipid accumulation depends on SREBP-1c. This supports the thesis that *MALAT1* plays a role in hepatic steatosis and insulin resistance. In conclusion, *MALAT1* induces hepatic lipid accumulation and insulin resistance by increasing the expression of *SREBP-1c* and target genes. This study suggests that *MALAT1* inhibition may have potential in the treatment of obesity and type 2 diabetes.

To build on our previous study [23], in which *MALAT1* was positively correlated with fat accumulation in the subcutaneous fat tissue of a native fatty Polish pig breed, further work is needed to establish the role that this lncRNA plays during this process in pigs, whether it is in adipogenesis or the proliferation process of primary adipose cells. However, the evidence presented in this review highlights a wide range of *MALAT1* functions in the generation of fat tissue, and we see a gap in the studies of *MALAT1* using other lab animals.

## 8. Future Perspective

*MALAT1* is a lncRNA that is expressed in multiple tissues and plays a significant role in critical molecular processes associated with the cell cycle, cancer, or hypoxia. Moreover, the evidence suggests that it is involved in numerous pathophysiological processes associated with many disorders or diseases in vascular or neurological systems and in cancer biology. Though *MALAT1* executes its function during gene expression in the alternative splicing and transcriptional and post-transcriptional regulation of many genes, its simple silencing or overexpression seem to be promising as a target for disease contradiction. However, *MALAT1* is expressed in almost all human tissues and has physiological functions in cells that are non-associated with pathological states. Therefore, it seems more effective to aim to limit RNA–protein interactions, which play a significant role during, for example, cancer progression. On the other hand, *MALAT1* is expressed as multiple isoforms dependent on the tissue, and these isoforms often have specific functions. Therefore, the deepening of research in the context of *MALAT1* isoforms may reveal a therapeutic target against cancer.

On the other hand, obesity is treated as a civilization disease, and we are still searching for gene targets for drugs that can be used to prevent this disorder. Currently, investigating individual genotypes makes it possible to estimate the predisposition to obesity occurrence and, based on this information and nutrigenetics, adjust the appropriate diet to individuals. Therefore, research should also focus on this aspect of *MALAT1,* searching of genetic markers association with regulation of adipogenesis and proliferation of primary fat cells, a mechanism related to fat deposition. Additionally, extending our knowledge of the new functions of lncRNAs delivers new possibilities for testing these interesting molecules.

As a final suggestion, farm animals are successfully used as study models in investigations aiming to find molecular mechanisms associated with fat deposition; therefore, the role of *MALAT1* in this context may be examined using a different model than humans or small lab animals. Based on our experience, we recommend pigs as an animal model.

## Data Availability

No new data were created or analyzed in this study. Data sharing is not applicable to this article.

## References

[1] Mattick J.S., Amaral P.P., Carninci P., Carpenter S., Chang H.Y., Chen L.L., Chen R., Dean C., Dinger M.E., Fitzgerald K.A. (2023). Long non-coding RNAs: Definitions, functions, challenges and recommendations. Nat. Rev. Mol. Cell Biol..

[2] Patraquim P., Magny E.G., Pueyo J.I., Platero A.I., Couso J.P. (2022). Translation and natural selection of micropeptides from long non-canonical RNAs. Nat. Commun..

[3] Ma L., Zhang Z. (2023). The contribution of databases towards understanding the universe of long non-coding RNAs. Nat. Rev. Mol. Cell Biol..

[4] Chen Y., Long W., Yang L., Zhao Y., Wu X., Li M., Du F., Chen Y., Yang Z., Wen Q. (2021). Functional Peptides Encoded by Long Non-Coding RNAs in Gastrointestinal Cancer. Front. Oncol..

[5] Zhang Y., Wang X., Hu C., Yi H. (2023). Shiny transcriptional junk: lncRNA-derived peptides in cancers and immune responses. Life Sci..

[6] Necsulea A., Soumillon M., Warnefors M., Liechti A., Daish T., Zeller U., Baker J.C., Grützner F., Kaessmann H. (2014). The evolution of lncRNA repertoires and expression patterns in tetrapods. Nature.

[7] Hon C.C., Ramilowski J.A., Harshbarger J., Bertin N., Rackham O.J.L., Gough J., Denisenko E., Schmeier S., Poulsen T.M., Severin J. (2017). An atlas of human long non-coding RNAs with accurate 5′ ends. Nature.

[8] Haerty W., Ponting C.P. (2013). Mutations within lncRNAs are effectively selected against in fruitfly but not in human. Genome Biol..

[9] Ponjavic J., Ponting C.P., Lunter G. (2007). Functionality or transcriptional noise? Evidence for selection within long noncoding RNAs. Genome Res..

[10] Kapusta A., Feschotte C. (2014). Volatile evolution of long noncoding RNA repertoires: Mechanisms and biological implications. Trends Genet..

[11] Liu L., Li Z., Liu C., Zou D., Li Q., Feng C., Jing W., Luo S., Zhang Z., Ma L. (2022). LncRNAWiki 2.0: A knowledgebase of human long non-coding RNAs with enhanced curation model and database system. Nucleic Acids Res..

[12] Böhmdorfer G., Wierzbicki A.T. (2015). Control of Chromatin Structure by Long Noncoding RNA. Trends Cell Biol..

[13] Cesana M., Cacchiarelli D., Legnini I., Santini T., Sthandier O., Chinappi M., Tramontano A., Bozzoni I. (2011). A long noncoding RNA controls muscle differentiation by functioning as a competing endogenous RNA. Cell.

[14] Ørom U.A., Derrien T., Beringer M., Gumireddy K., Gardini A., Bussotti G., Lai F., Zytnicki M., Notredame C., Huang Q. (2010). Long noncoding RNAs with enhancer like function in human cells. Cell.

[15] Statello L., Guo C.J., Chen L.L., Huarte M. (2020). Gene regulation by long non-coding RNAs and its biological functions. Nat. Rev. Mol. Cell Biol..

[16] Smith J.E., Alvarez-Dominguez J.R., Kline N., Huynh N.J., Geisler S., Hu W., Coller J., Baker K.E. (2014). Translation of small open reading frames within unannotated RNA transcripts in Saccharomyces cerevisiae. Cell Rep..

[17] Hutchinson J.N., Ensminger A.W., Clemson C.M., Lynch C.R., Lawrence J.B., Chess A. (2007). A screen for nuclear transcripts identifies two linked noncoding RNAs associated with SC35 splicing domains. BMC Genom..

[18] Ji P., Diederichs S., Wang W., Böing S., Metzger R., Schneider P.M., Tidow N., Brandt B., Buerger H., Bulk E. (2003). MALAT-1, a novel noncoding RNA, and thymosin β4 predict metastasis and survival in early-stage non-small cell lung cancer. Oncogene.

[19] Zhang X., Hamblin M.H., Yin K.J. (2017). The long noncoding RNA Malat1: Its physiological and pathophysiological functions. RNA Biol..

[20] Masoumi F., Ghorbani S., Talebi F., Branton W.G., Rajaei S., Power C., Noorbakhsh F. (2019). Malat1 long noncoding RNA regulates inflammation and leukocyte differentiation in experimental autoimmune encephalomyelitis. J. Neuroimmunol..

[21] Gao Q., Wang Y. (2020). Long noncoding RNA MALAT1 regulates apoptosis in ischemic stroke by sponging miR-205-3p and modulating PTEN expression. Am. J. Transl. Res..

[22] Wang L., Li S., Stone S.S., Liu N., Gong K., Ren C., Sun K., Zhang C., Shao G. (2022). The Role of the lncRNA MALAT1 in Neuroprotection against Hypoxic/Ischemic Injury. Biomolecules.

[23] Piórkowska K., Żukowski K., Ropka-Molik K., Tyra M. (2022). New long-non coding RNAs related to fat deposition based on pig model. Ann. Anim. Sci..

[24] Yang Y., Zhou R., Zhu S., Li X., Li H., Yu H., Li K. (2017). Systematic Identification and Molecular Characteristics of Long Noncoding RNAs in Pig Tissues. Biomed. Res. Int..

[25] Wilusz J.E., Freier S.M., Spector D.L. (2008). 3′ end processing of a long nuclear-retained noncoding RNA yields a tRNA-like cytoplasmic RNA. Cell.

[26] Clemson C.M., Hutchinson J.N., Sara S.A., Ensminger A.W., Fox A.H., Chess A., Lawrence J.B. (2009). An architectural role for a nuclear noncoding RNA: NEAT1 RNA is essential for the structure of paraspeckles. Mol. Cell.

[27] Brown J.A., Bulkley D., Wang J., Valenstein M.L., Yario T.A., Steitz T.A., Steitz J.A. (2014). Structural insights into the stabilization of MALAT1 noncoding RNA by a bipartite triple helix. Nat. Struct. Mol. Biol..

[28] Clark M.B., Johnston R.L., Inostroza-Ponta M., Fox A.H., Fortini E., Moscato P., Dinger M.E., Mattick J.S. (2012). Genome-wide analysis of long noncoding RNA stability. Genome Res..

[29] Arun G., Aggarwal D., Spector D.L. (2020). MALAT1 Long Non-Coding RNA: Functional Implications. Non-Coding RNA.

[30] Lin S., Lin Y., Nery J.R., Urich M.A., Breschi A., Davis C.A., Dobin A., Zaleski C., Beer M.A., Chapman W.C. (2014). Comparison of the transcriptional landscapes between human and mouse tissues. Proc. Natl. Acad. Sci. USA.

[31] Huang Z., Huang L., Shen S., Li J., Lu H., Mo W., Dang Y., Luo D., Chen G., Feng Z. (2015). Sp1 cooperates with Sp3 to upregulate MALAT1 expression in human hepatocellular carcinoma. Oncol. Rep..

[32] Zhao Y., Yang Y., Trovik J., Sun K., Zhou L., Jiang P., Lau T.S., Hoivik E.A., Salvesen H.B., Sun H. (2014). A novel wnt regulatory axis in endometrioid endometrial cancer. Cancer Res..

[33] Wenger R., Lelli A., Nolan K., Santambrogio S., Marti H., Hoogewijs D., Frew I., Goncalves A.F., Schonenberger M., Guinot A. (2015). Induction of long noncoding RNA MALAT1 in hypoxic mice. Hypoxia.

[34] Sallé-Lefort S., Miard S., Nolin M.A., Boivin L., Paré M.È., Debigaré R., Picard F. (2016). Hypoxia upregulates Malat1 expression through a CaMKK/AMPK/HIF-1α axis. Int. J. Oncol..

[35] Luo F., Sun B., Li H., Xu Y., Liu Y., Liu X., Lu L., Li J., Wang Q., Wei S. (2016). A MALAT1/HIF-2α feedback loop contributes to arsenite carcinogenesis. Oncotarget.

[36] Fuschi P., Carrara M., Voellenkle C., Garcia-Manteiga J.M., Righini P., Maimone B., Sangalli E., Villa F., Specchia C., Picozza M. (2017). Central role of the p53 pathway in the noncoding-RNA response to oxidative stress. Aging.

[37] Ma X.Y., Wang J.H., Wang J.L., Ma C.X., Wang X.C., Liu F.S. (2015). Malat1 as an evolutionarily conserved lncRNA, plays a positive role in regulating proliferation and maintaining undifferentiated status of early-stage hematopoietic cells. BMC Genom..

[38] Sun H., Lin D.C., Cao Q., Pang B., Gae D.D., Lee V.K.M., Lim H.J., Doan N., Said J.W., Gery S. (2017). Identification of a Novel SYK/c-MYC/MALAT1 Signaling Pathway and Its Potential Therapeutic Value in Ewing Sarcoma. Clin. Cancer Res..

[39] Sun D., Li X., He Y., Li W., Wang Y., Wang H., Jiang S., Xin Y. (2016). YAP1 enhances cell proliferation, migration, and invasion of gastric cancer in vitro and in vivo. Oncotarget.

[40] Amodio N., Stamato M.A., Juli G., Morelli E., Fulciniti M., Manzoni M., Taiana E., Agnelli L., Cantafio M.E.G., Romeo E. (2018). Drugging the lncRNA MALAT1 via LNA gapmeR ASO inhibits gene expression of proteasome subunits and triggers anti-multiple myeloma activity. Leukemia.

[41] Radhakrishnan S.K., Lee C.S., Young P., Beskow A., Chan J.Y., Deshaies R.J. (2010). Transcription factor Nrf1 mediates the proteasome recovery pathway after proteasome inhibition in mammalian cells. Mol. Cell.

[42] Manasanch E.E., Orlowski R.Z. (2017). Proteasome inhibitors in cancer therapy. Nat. Rev. Clin. Oncol..

[43] Yen C.H., Hsu C.M., Hsiao S.Y., Hsiao H.H. (2020). Pathogenic Mechanisms of Myeloma Bone Disease and Possible Roles for NRF2. Int. J. Mol. Sci..

[44] Kuo I.Y., Wu C.C., Chang J.M., Huang Y.L., Lin C.H., Yan J.J., Sheu B.S., Lu P.J., Chang W.L., Lai W.W. (2014). Low SOX17 expression is a prognostic factor and drives transcriptional dysregulation and esophageal cancer progression. Int. J. Cancer.

[45] Wang X., Li M., Wang Z., Han S., Tang X., Ge Y., Zhou L., Zhou C., Yuan Q., Yang M. (2014). Silencing of Long Noncoding RNA MALAT1 by miR-101 and miR-217 Inhibits Proliferation, Migration, and Invasion of Esophageal Squamous Cell Carcinoma Cells. J. Biol. Chem..

[46] Koshimizu T., Fujiwara Y., Sakai N., Shibata K., Tsuchiya H. (2010). Oxytocin stimulates expression of a noncoding RNA tumor marker in a human neuroblastoma cell line. Life Sci..

[47] Fan Y., Shen B., Tan M., Mu X., Qin Y., Zhang F., Liu Y. (2014). TGF-β-induced upregulation of malat1 promotes bladder cancer metastasis by associating with suz12. Clin. Cancer Res..

[48] Li C., Chang L., Chen Z., Liu Z., Wang Y., Ye Q. (2017). The role of lncRNA MALAT1 in the regulation of hepatocyte proliferation during liver regeneration. Int. J. Mol. Med..

[49] Zhang D., Xue J., Peng F. (2022). The regulatory activities of MALAT1 in the development of bone and cartilage diseases. Front. Endocrinol..

[50] Zhou L., Xu D.-Y., Sha W.-G., Shen L., Lu G.-Y. (2018). Long non-coding RNA MALAT1 interacts with transcription factor Foxo1 to regulate SIRT1 transcription in high glucose-induced HK-2 cells injury. Biochem. Biophys. Res. Commun..

[51] Leucci E., Patella F., Waage J., Holmstrøm K., Lindow M., Porse B., Kauppinen S., Lund A.H. (2013). microRNA-9 targets the long non-coding RNA MALAT1 for degradation in the nucleus. Sci. Rep..

[52] Vermeulen K., Berneman Z.N., Van Bockstaele D.R. (2003). Cell cycle and apoptosis. Cell Prolif..

[53] Tripathi V., Shen Z., Chakraborty A., Giri S., Freier S.M., Wu X., Zhang Y., Gorospe M., Prasanth S.G., Lal A. (2013). Long noncoding RNA MALAT1 controls cell cycle progression by regulating the expression of oncogenic transcription factor B-MYB. PLoS Genet..

[54] Zhang C., Wang J., Guo L., Peng M. (2021). Long non-coding RNA MALAT1 regulates cell proliferation, invasion and apoptosis by modulating the Wnt signaling pathway in squamous cell carcinoma. Am. J. Transl. Res..

[55] Dai X., Liu L., Liang Z., Guo K., Xu S., Wang H. (2019). Silencing of lncRNA MALAT1 inhibits cell cycle progression via androgen receptor signaling in prostate cancer cells. Pathol. Res. Pract..

[56] Sadasivam S., Duan S., DeCaprio J.A. (2012). The MuvB complex sequentially recruits B-Myb and FoxM1 to promote mitotic gene expression. Genes Dev..

[57] Yang F., Yi F., Han X., Du Q., Liang Z. (2013). MALAT-1 interacts with hnRNP C in cell cycle regulation. FEBS Lett..

[58] Hou J., Zhang G., Wang X., Wang Y., Wang K. (2023). Functions and mechanisms of lncRNA MALAT1 in cancer chemotherapy resistance. Biomark. Res..

[59] Du B., Wang J., Zang S., Mao X., Du Y. (2020). Long non-coding RNA MALAT1 suppresses the proliferation and migration of endothelial progenitor cells in deep vein thrombosis by regulating the Wnt/β-catenin pathway. Exp. Ther. Med..

[60] Hajibabaei S., Nafissi N., Azimi Y., Mahdian R., Rahimi-Jamnani F., Valizadeh V., Rafiee M.H., Azizi M. (2023). Targeting long non-coding RNA MALAT1 reverses cancerous phenotypes of breast cancer cells through microRNA-561-3p/TOP2A axis. Sci. Rep..

[61] Hu L., Wu Y., Tan D., Meng H., Wang K., Bai Y., Yang K. (2015). Up-regulation of long noncoding RNA MALAT1 contributes to proliferation and metastasis in esophageal squamous cell carcinoma. J. Exp. Clin. Cancer Res..

[62] Guffanti A., Iacono M., Pelucchi P., Kim N., Soldà G., Croft L.J., Taft R.J., Rizzi E., Askarian-Amiri M., Bonnal R.J. (2009). A transcriptional sketch of a primary human breast cancer by 454 deep sequencing. BMC Genom..

[63] Chen Q., Su Y., He X., Zhao W., Wu C., Zhang W., Si X., Dong B., Zhao L., Gao Y. (2016). Plasma long non-coding RNA MALAT1 is associated with distant metastasis in patients with epithelial ovarian cancer. Oncol. Lett..

[64] Shi D., Zhang Y., Lu R., Zhang Y. (2018). The long non-coding RNA MALAT1 interacted with miR-218 modulates choriocarcinoma growth by targeting Fbxw8. Biomed. Pharmacother..

[65] Wang J., Su L., Chen X., Li P., Cai Q., Yu B., Liu B., Wu W., Zhu Z. (2014). MALAT1 promotes cell proliferation in gastric cancer by recruiting SF2/ASF. Biomed. Pharmacother..

[66] Qi Y., Ooi H.S., Wu J., Chen J., Zhang X., Tan S., Yu Q., Li Y.Y., Kang Y., Li H. (2016). MALAT1 long ncRNA promotes gastric cancer metastasis by suppressing PCDH10. Oncotarget.

[67] Yang M.H., Hu Z.Y., Xu C., Xie L.Y., Wang X.Y., Chen S.Y., Li Z.G. (2015). MALAT1 promotes colorectal cancer cell proliferation/migration/invasion via PRKA kinase anchor protein 9. Biochim. Biophys. Acta.

[68] Zhang T.H., Liang L.Z., Liu X.L., Wu J.N., Su K., Chen J.Y., Zheng Q.Y., Huang H.Z., Liao G.Q. (2017). Long non-coding RNA MALAT1 interacts with miR-124 and modulates tongue cancer growth by targeting JAG1. Oncol. Rep..

[69] Tan X., Huang Z., Li X. (2017). Long Non-Coding RNA MALAT1 Interacts With miR-204 to Modulate Human Hilar Cholangiocarcinoma Proliferation, Migration, and Invasion by Targeting CXCR4. J. Cell. Biochem..

[70] Han Y., Liu Y., Nie L., Gui Y., Cai Z. (2013). Inducing cell proliferation inhibition, apoptosis, and motility reduction by silencing long noncoding ribonucleic acid metastasis-associated lung adenocarcinoma transcript 1 in urothelial carcinoma of the bladder. Urology.

[71] Liu X., Lv R., Zhang L., Xu G., Bi J., Gao F., Zhang J., Xue F., Wang F., Wu Y. (2016). Long noncoding RNA expression profile of infantile hemangioma identified by microarray analysis. Tumour Biol..

[72] Huang J.K., Ma L., Song W.H., Lu B.Y., Huang Y.B., Dong H.M., Ma X.K., Zhu Z.Z., Zhou R. (2017). LncRNA-MALAT1 Promotes Angiogenesis of Thyroid Cancer by Modulating Tumor-Associated Macrophage FGF2 Protein Secretion. J. Cell. Biochem..

[73] Puthanveetil P., Chen S., Feng B., Gautam A., Chakrabarti S. (2015). Long non-coding RNA MALAT1 regulates hyperglycaemia induced inflammatory process in the endothelial cells. J. Cell. Mol. Med..

[74] Yan B., Tao Z.F., Li X.M., Zhang H., Yao J., Jiang Q. (2014). Aberrant expression of long noncoding RNAs in early diabetic retinopathy. Investig. Ophthalmol. Vis. Sci..

[75] Zhou R.M., Wang X.Q., Yao J., Shen Y., Chen S.N., Yang H., Jiang Q., Yan B. (2015). Identification and characterization of proliferative retinopathy-related long noncoding RNAs. Biochem. Biophys. Res. Commun..

[76] Yan C., Chen J., Chen N. (2016). Long noncoding RNA MALAT1 promotes hepatic steatosis and insulin resistance by increasing nuclear SREBP-1c protein stability. Sci. Rep..

[77] Zhuo Y., Zeng Q., Zhang P., Li G., Xie Q., Cheng Y. (2017). Functional polymorphism of lncRNA MALAT1 contributes to pulmonary arterial hypertension susceptibility in Chinese people. Clin. Chem. Lab. Med..

[78] Wang D., Ding L., Wang L., Zhao Y., Sun Z., Karnes R.J., Zhang J., Huang H. (2015). LncRNA MALAT1 enhances oncogenic activities of EZH2 in castration-resistant prostate cancer. Oncotarget.

[79] Zheng X., Ren J., Peng B., Ye J., Wu X., Zhao W., Li Y., Chen R., Gong X., Bai C. (2020). MALAT1 overexpression promotes the growth of colon cancer by repressing β-catenin degradation. Cell. Signal..

[80] Kim S.H., Kim S.H., Yang W.I., Kim S.J., Yoon S.O. (2017). Association of the long non-coding RNA MALAT1 with the polycomb repressive complex pathway in T and NK cell lymphoma. Oncotarget.

[81] Arratia F., Fierro C., Blanco A., Fuentes S., Nahuelquen D., Montecino M., Rojas A., Aguilar R. (2023). Selective Concurrence of the Long Non-Coding RNA MALAT1 and the Polycomb Repressive Complex 2 to Promoter Regions of Active Genes in MCF7 Breast Cancer Cells. Curr. Issues Mol. Biol..

[82] Liang J., Liang L., Ouyang K., Li Z., Yi X. (2017). MALAT1 induces tongue cancer cells’ EMT and inhibits apoptosis through Wnt/β-catenin signaling pathway. J. Oral Pathol. Med..

[83] Li Z., Wu Y., Ma C., Nie S., Mao X., Shi Y. (2011). Down-regulation of c-Myc expression inhibits the invasion of bile duct carcinoma cells. Cell Biol. Int..

[84] Zhang J., Gill A.J.M., Issacs J.D., Atmore B., Johns A., Delbridge L.W., Lai R., McMullen T.P.W. (2012). The Wnt/β-catenin pathway drives increased cyclin D1 levels in lymph node metastasis in papillary thyroid cancer. Hum. Pathol..

[85] Yang X., Du X., Sun L., Zhao X., Zhu J., Li G., Tian J., Li X., Wang Z. (2019). SULT2B1b promotes epithelial-mesenchymal transition through activation of the β-catenin/MMP7 pathway in hepatocytes. Biochem. Biophys. Res. Commun..

[86] Zeilstra J., Joosten S.P.J., Dokter M., Verwiel E., Spaargaren M., Pals S.T. (2008). Deletion of the WNT target and cancer stem cell marker CD44 in Apc(Min/+) mice attenuates intestinal tumorigenesis. Cancer Res..

[87] Jin Y., Feng S.J., Qiu S., Shao N., Zheng J.H. (2017). LncRNA MALAT1 promotes proliferation and metastasis in epithelial ovarian cancer via the PI3K-AKT pathway. Eur. Rev. Med. Pharmacol. Sci..

[88] Peng N., He J., Li J., Huang H., Huang W., Liao Y., Zhu S. (2020). Long noncoding RNA MALAT1 inhibits the apoptosis and autophagy of hepatocellular carcinoma cell by targeting the microRNA-146a/PI3K/Akt/mTOR axis. Cancer Cell Int..

[89] Huang J.L., Liu W., Tian L.H., Chai T.T., Liu Y., Feng Z., Fu H.Y., Zhou H.R., Shen J.Z. (2017). Upregulation of long non-coding RNA MALAT-1 confers poor prognosis and influences cell proliferation and apoptosis in acute monocytic leukemia. Oncol. Rep..

[90] Guo F., Li Y., Liu Y., Wang J., Li Y., Li G. (2010). Inhibition of metastasis-associated lung adenocarcinoma transcript 1 in CaSki human cervical cancer cells suppresses cell proliferation and invasion. Acta Biochim. Biophys. Sin..

[91] Ji D.G., Guan L.Y., Luo X., Ma F., Yang B., Liu H.Y. (2018). Inhibition of MALAT1 sensitizes liver cancer cells to 5-flurouracil by regulating apoptosis through IKKα/NF-κB pathway. Biochem. Biophys. Res. Commun..

[92] Si Y., Yang Z., Ge Q., Yu L., Yao M., Sun X., Ren Z., Ding C. (2019). Long non-coding RNA Malat1 activated autophagy, hence promoting cell proliferation and inhibiting apoptosis by sponging miR-101 in colorectal cancer. Cell. Mol. Biol. Lett..

[93] Xie H., Liao X., Chen Z., Fang Y., He A., Zhong Y., Gao Q., Xiao H., Li J., Huang W. (2017). LncRNA MALAT1 Inhibits Apoptosis and Promotes Invasion by Antagonizing miR-125b in Bladder Cancer Cells. J. Cancer.

[94] Sun Y., Qin B. (2018). Long noncoding RNA MALAT1 regulates HDAC4-mediated proliferation and apoptosis via decoying of miR-140-5p in osteosarcoma cells. Cancer Med..

[95] Wang R., Lu X., Yu R. (2020). Lncrna malat1 promotes emt process and cisplatin resistance of oral squamous cell carcinoma via pi3k/akt/m-tor signal pathway. Onco. Targets. Ther..

[96] Zhang Z.C., Tang C., Dong Y., Zhang J., Yuan T., Tao S.C., Li X.L. (2017). Targeting the long noncoding RNA MALAT1 blocks the pro-angiogenic effects of osteosarcoma and suppresses tumour growth. Int. J. Biol. Sci..

[97] Spector D.L., Lamond A.I. (2011). Nuclear Speckles. Cold Spring Harb. Perspect. Biol..

[98] Malakar P., Shilo A., Mogilevsky A., Stein I., Pikarsky E., Nevo Y., Benyamini H., Elgavish S., Zong X., Prasanth K.V. (2017). Long Noncoding RNA MALAT1 Promotes Hepatocellular Carcinoma Development by SRSF1 Upregulation and mTOR Activation. Cancer Res..

[99] Miao H., Wu F., Li Y., Qin C., Zhao Y., Xie M., Dai H., Yao H., Cai H., Wang Q. (2022). MALAT1 modulates alternative splicing by cooperating with the splicing factors PTBP1 and PSF. Sci. Adv..

[100] Wang Y., Zhang Y., Hu K., Qiu J., Hu Y., Zhou M., Zhang S. (2020). Elevated long noncoding RNA MALAT-1 expression is predictive of poor prognosis in patients with breast cancer: A meta-analysis. Biosci. Rep..

[101] Schmidt L.H., Spieker T., Koschmieder S., Humberg J., Jungen D., Bulk E., Hascher A., Wittmer D., Marra A., Hillejan L. (2011). The Long Noncoding MALAT-1 RNA Indicates a Poor Prognosis in Non-small Cell Lung Cancer and Induces Migration and Tumor Growth. J. Thorac. Oncol..

[102] Ma K.X., Wang H.J., Li X.R., Li T., Su G., Yang P., Wu J.W. (2015). Long noncoding RNA MALAT1 associates with the malignant status and poor prognosis in glioma. Tumor Biol..

[103] Hong J.H., Jin E.H., Chang I.A., Kang H., Lee S.I., Sung J.K. (2020). Association of long noncoding RNA MALAT1 polymorphisms with gastric cancer risk in Korean individuals. Mol. Genet. Genom. Med..

[104] Wen J., Chen L., Tian H., Li J., Zhang M., Cao Q., Zhang W., Chen S., Shi L. (2019). Effect of MALAT1 Polymorphisms on Papillary Thyroid Cancer in a Chinese Population. J. Cancer.

[105] Yuan L.T., Chang J.H., Lee H.L., Yang Y.C., Su S.C., Lin C.L., Yang S.F., Chien M.H. (2019). Genetic Variants of lncRNA MALAT1 Exert Diverse Impacts on the Risk and Clinicopathologic Characteristics of Patients with Hepatocellular Carcinoma. J. Clin. Med..

[106] Zheng L., Rong L., Cheng Z. (2020). Association between LncRNA MALAT1 Polymorphisms and Cancer Risk: A Meta-Analysis Based on7007 Cases and 8791 Controls. https://www.researchsquare.com/article/rs-42022/v1.

[107] Wu S., Sun H., Wang Y., Yang X., Meng Q., Yang H., Zhu H., Tang W., Li X., Aschner M. (2019). MALAT1 rs664589 Polymorphism Inhibits Binding to miR-194-5p, Contributing to Colorectal Cancer Risk, Growth, and Metastasis. Cancer Res..

[108] Zhang P., Wu S., He Y., Li X., Zhu Y., Lin X., Chen L., Zhao Y., Niu L., Zhang S. (2022). LncRNA-Mediated Adipogenesis in Different Adipocytes. Int. J. Mol. Sci..

[109] Powell-Wiley T.M., Poirier P., Burke L.E., Després J.P., Gordon-Larsen P., Lavie C.J., Lear S.A., Ndumele C.E., Neeland I.J., Sanders P. (2021). Obesity and Cardiovascular Disease: A Scientific Statement From the American Heart Association. Circulation.

[110] Chandrasekaran P., Weiskirchen R. (2024). The Role of Obesity in Type 2 Diabetes Mellitus—An Overview. Int. J. Mol. Sci..

[111] Shariq O.A., Mckenzie T.J. (2020). Obesity-related hypertension: A review of pathophysiology, management, and the role of metabolic surgery. Gland Surg..

[112] Avgerinos K.I., Spyrou N., Mantzoros C.S., Dalamaga M. (2019). Obesity and cancer risk: Emerging biological mechanisms and perspectives. Metabolism.

[113] Wijesinghe S.N., Nicholson T., Tsintzas K., Jones S.W. (2021). Involvements of long noncoding RNAs in obesity-associated inflammatory diseases. Obes. Rev..

[114] Sun Y., Chen X., Qin J., Liu S., Zhao R., Yu T., Chu G., Yang G., Pang W. (2018). Comparative Analysis of Long Noncoding RNAs Expressed during Intramuscular Adipocytes Adipogenesis in Fat-Type and Lean-Type Pigs. J. Agric. Food Chem..

[115] Yu L., Tai L., Zhang L., Chu Y., Li Y., Zhou L. (2017). Comparative analyses of long non-coding RNA in lean and obese pig. Oncotarget.

[116] Sun Y., Cai R., Wang Y., Zhao R., Qin J., Pang W. (2020). A newly identified LNcRNA LncIMF4 controls adipogenesis of porcine intramuscular preadipocyte through attenuating autophagy to inhibit lipolysis. Animals.

[117] Carter S., Miard S., Boivin L., Sallé-Lefort S., Picard F. (2018). Loss of Malat1 does not modify age- or diet-induced adipose tissue accretion and insulin resistance in mice. PLoS ONE.

[118] Kong X., Patel N.A., Chalfant C.E., Cooper D.R. (2023). Ceramide synthesis regulates biogenesis and packaging of exosomal MALAT1 from adipose derived stem cells, increases dermal fibroblast migration and mitochondrial function. Cell Commun. Signal..

[119] Han J., Shen L., Zhan Z., Liu Y., Zhang C., Guo R., Luo Y., Xie Z., Feng Y., Wu G. (2021). The long noncoding RNA MALAT1 modulates adipose loss in cancer-associated cachexia by suppressing adipogenesis through PPAR-γ. Nutr. Metab..

[120] Patel R.S., Carter G., El Bassit G., Patel A.A., Cooper D.R., Murr M., Patel N.A. (2016). Adipose-derived stem cells from lean and obese humans show depot specific differences in their stem cell markers, exosome contents and senescence: Role of protein kinase C delta (PKCδ) in adipose stem cell niche. Stem Cell Investig..

[121] Ebrahimi R., Toolabi K., Jannat Ali Pour N., Mohassel Azadi S., Bahiraee A., Zamani-Garmsiri F., Emamgholipour S. (2020). Adipose tissue gene expression of long non-coding RNAs; MALAT1, TUG1 in obesity: Is it associated with metabolic profile and lipid homeostasis-related genes expression?. Diabetol. Metab. Syndr..

[122] Rasaei N., Gholami F., Samadi M., Shiraseb F., Khadem A., Yekaninejad M.S., Emamgholipour S., Mirzaei K. (2024). The interaction between MALAT1 and TUG1 with dietary fatty acid quality indices on visceral adiposity index and body adiposity index. Sci. Rep..

[123] Liu L., Tan L., Yao J., Yang L. (2020). Long non-coding RNA MALAT1 regulates cholesterol accumulation in ox-LDL-induced macrophages via the microRNA-17-5p/ABCA1 axis. Mol. Med. Rep..

[124] Van Solingen C., Scacalossi K.R., Moore K.J. (2018). Long noncoding RNAs in lipid metabolism. Curr. Opin. Lipidol..

